# Association between sunlight exposure and risk of rheumatic diseases: a prospective cohort study in the UK

**DOI:** 10.3389/fpubh.2026.1873382

**Published:** 2026-09-14

**Authors:** Yang Pu, Jingru Chen, Linpeng Zhou, Yangchang Zhang, Yihan Zhi, Lingli Ding, Yu Gan, Li Chen, Chengyin Li, Bin Wu

**Affiliations:** 1 School of General Education, Chongqing College of Traditional Chinese Medicine, Chongqing, China; 2 Department of Rheumatology and Immunology, The First Affiliated Hospital of Chongqing College of Traditional Chinese Medicine, Chongqing, China; 3 School of Clinical Medicine, Chengdu University of Traditional Chinese Medicine, Chengdu, China; 4 National Health Commission Key Laboratory of Reproductive Health, School of Public Health, Institute of Child and Adolescent Health, Peking University, Beijing, China

**Keywords:** gout, outdoor light exposure, prospective cohort, restricted cubic spline, rheumatoid arthritis, systemic lupus erythematosus

## Abstract

**Objective:**

To investigate the relationships between sunlight exposure and the risk of three major rheumatic diseases (systemic lupus erythematosus [SLE], rheumatoid arthritis [RA], and gout) in a large-scale prospective cohort.

**Methods:**

Data were derived from the UK Biobank (a nationwide prospective cohort in the United Kingdom), including 363,873 participants followed up from 2006 to 2022. Sunlight exposure was assessed by self-reported average daily outdoor time in summer and winter. Incident SLE, RA, and gout were identified using International Classification of Diseases, Tenth Revision (ICD-10) codes from linked hospital records. Multivariate Cox proportional hazards models, restricted cubic splines, subgroup analyses, and sensitivity analyses were performed to evaluate associations.

**Results:**

During the 13-year follow-up, 335 SLE, 5,591 RA, and 7,485 gout cases were ascertained. Compared with sunlight exposure of 2.5 h/day, both lower and higher sunlight exposure were associated with increased risks of SLE (< 2.5 h/day: HR = 1.71, 95% CI:1.13, 2.59; >2.5 h/day: HR = 1.75, 95% CI:1.17, 2.63). We also observed increased risk associations of higher sunlight exposure with RA (HR = 1.15, 95% CI:1.06, 1.26) and gout (HR = 1.09, 95% CI:1.02, 1.18). Subgroup analyses suggested an age-related difference in the association between sunlight exposure and gout after Bonferroni correction (adjusted *p* value = 0.014).

**Conclusion:**

Sunlight exposure was associated with disease-specific risks of SLE, RA, and gout.

## Highlights

- Sunlight exposure was associated with distinct risk patterns across SLE, RA, and gout in a large-scale prospective cohort.
- Both lower and higher sunlight exposure were associated with increased SLE risk, whereas the associations with RA and gout were modest and should be interpreted cautiously.

## Introduction

Sunlight exposure represents one of the most pervasive environmental stimuli encountered throughout human life, yet its long-term effects on immune-mediated diseases remain incompletely understood (1). Beyond its classical role as a source of cutaneous photodamage, advances in photoimmunology have revealed that ultraviolet radiation (UVR) acts as an important regulator of immune homeostasis through interactions involving cellular stress responses, antigen processing, and inflammatory signaling. Importantly, the immunological effects of UVR are highly context dependent (2, 3). Depending on exposure characteristics and host susceptibility, UVR may induce immune regulatory responses under physiological conditions while promoting inflammatory and autoimmune processes in susceptible individuals (4, 5). Therefore, whether the consequences of sunlight exposure are shared across immune-mediated diseases or shaped by disease-specific pathogenic mechanisms remains an important unresolved question.

Among rheumatic diseases, SLE is one of the conditions most strongly associated with sunlight exposure (6). Photosensitivity is a well-recognized clinical hallmark of SLE, and UVR has been proposed to contribute to disease pathogenesis through mechanisms involving keratinocyte apoptosis, autoantigen release, and type I interferon activation (7–9). However, previous studies have predominantly focused on UV-induced photosensitivity, cutaneous manifestations, and disease activity among patients with established SLE (10, 11). Although ultraviolet exposure has been implicated as an environmental factor contributing to SLE susceptibility, evidence from prospective population-based studies evaluating habitual sunlight exposure in relation to incident SLE remains limited.

Beyond SLE, the role of sunlight exposure in other rheumatic diseases has received comparatively less attention. Rheumatic diseases encompass diverse immunopathological processes, and the biological effects of the same environmental exposure may vary depending on the underlying disease mechanisms. Rheumatoid arthritis (RA) and gout represent two distinct inflammatory models with different immunological foundations: RA is characterized by autoimmune-mediated adaptive immune dysregulation (12), whereas gout is primarily driven by innate immune activation triggered by monosodium urate crystal deposition (13). However, whether sunlight exposure influences the development of these diseases, and whether such effects differ from those observed in SLE, remains largely unknown.

Therefore, prospective studies evaluating multiple rheumatic diseases within a unified population framework are needed. Using data from the UK Biobank, we conducted a prospective cohort study to investigate the associations between sunlight exposure and incident SLE, RA, and gout. By comparing diseases with distinct immunological backgrounds within the same cohort, we aimed to determine whether sunlight exposure exhibits disease-specific associations across rheumatic disorders.

## Methods

### Study design

Data for this study were derived from the UK Biobank, a nationwide prospective cohort that enrolled approximately 500,000 adults from across the United Kingdom between 2006 and 2010. The cohort primarily comprised individuals aged 40 years and older at baseline, who were followed longitudinally through linked health records until death, withdrawal, or the end of follow-up. Extensive information was systematically collected through touchscreen questionnaires, nurse-led interviews, physical examinations, biological specimen collection, imaging assessments, and linkage to national health registries, thereby providing a rich resource for population-based research. Ethical approval was granted by the North West Multi-Centre Research Ethics Committee (number: 21/NW/0157), and all participants provided written informed consent.

### Assessment of sunlight exposure

Sunlight exposure was assessed based on self-reported daily time spent outdoors during typical summer and winter days. Data were collected at baseline (2006–2010) through touchscreen questionnaires, corresponding to fields 1050 (summer) and 1060 (winter) in the UK Biobank database. Participants were asked the question: “In a typical day in summer (winter), how many hours do you spend outdoors?” If the amount of time spent outdoors in summer (winter) varied substantially, participants were instructed to report the average daily time. For example, if a participant spent 1 h per day on weekdays and 4 h per day on weekends, the total weekly duration would be 13 h (5 + 8), corresponding to an average of approximately 2 h per day. The touchscreen questionnaire included no items on occupational or recreational sun exposure and no personal ultraviolet dosimetry was performed, so the exposure analyzed here represents self-reported time spent outdoors rather than a measured ultraviolet dose.

During data preprocessing, responses of missing or ambiguous values in summer (53,438) or winter (112,247) were removed, respectively. The average daily sunlight exposure duration was calculated as the mean of the reported summer and winter durations. To minimize the influence of extreme values on the analysis, participants with average daily sunlight exposure above the 95th percentile or below the 5th percentile were excluded.

### Covariates

A series of covariates were selected into our analyses, including age (< 65 years and ≥ 65 years), sex (Male and Female), ethnicity (White and Non-white), educational levels (College and Other levels), Body mass index (BMI) (kg/m^2^) (< 24, 24 ~ 27.9, ≥ 28), alcohol consumption (Yes and No), smoking status (Yes and No), household income (< £ 31,000 and ≥ £ 31,000), physical activity (Yes and No), dieting score (ranged from 0–7), sleep duration (≤ 7 h and > 7 h), ambient PM_2.5_ levels (μg/m^3^), skin color (White and Other), use of sun exposure protection (Yes and No). When performing regression analyses, missing values in continuous variables were imputed using the mean, whereas missing values in categorical variables were imputed using the mode. Use of sun protection was ascertained with a single baseline question and cut as a binary variable (yes versus no), which captures neither its type nor its frequency or adequacy.

### Outcome definition

Incident rheumatoid arthritis, systemic lupus erythematosus, and gout were ascertained from linked hospital admission records in the UK Biobank using International Classification of Diseases, Tenth Revision (ICD-10) codes. According to ICD-10, systemic lupus erythematosus was defined using 4 codes; rheumatoid arthritis was identified using 15 codes; gout was ascertained using 38 codes (Supplementary Table S1). Dates of death were obtained from the Office for National Statistics mortality register. Follow-up extended from baseline to the earliest of outcome diagnosis, death, or loss to follow-up and was censored on October 31, 2022. A detailed flow of participant inclusion and exclusion is presented in Figure 1.

**Figure 1:**
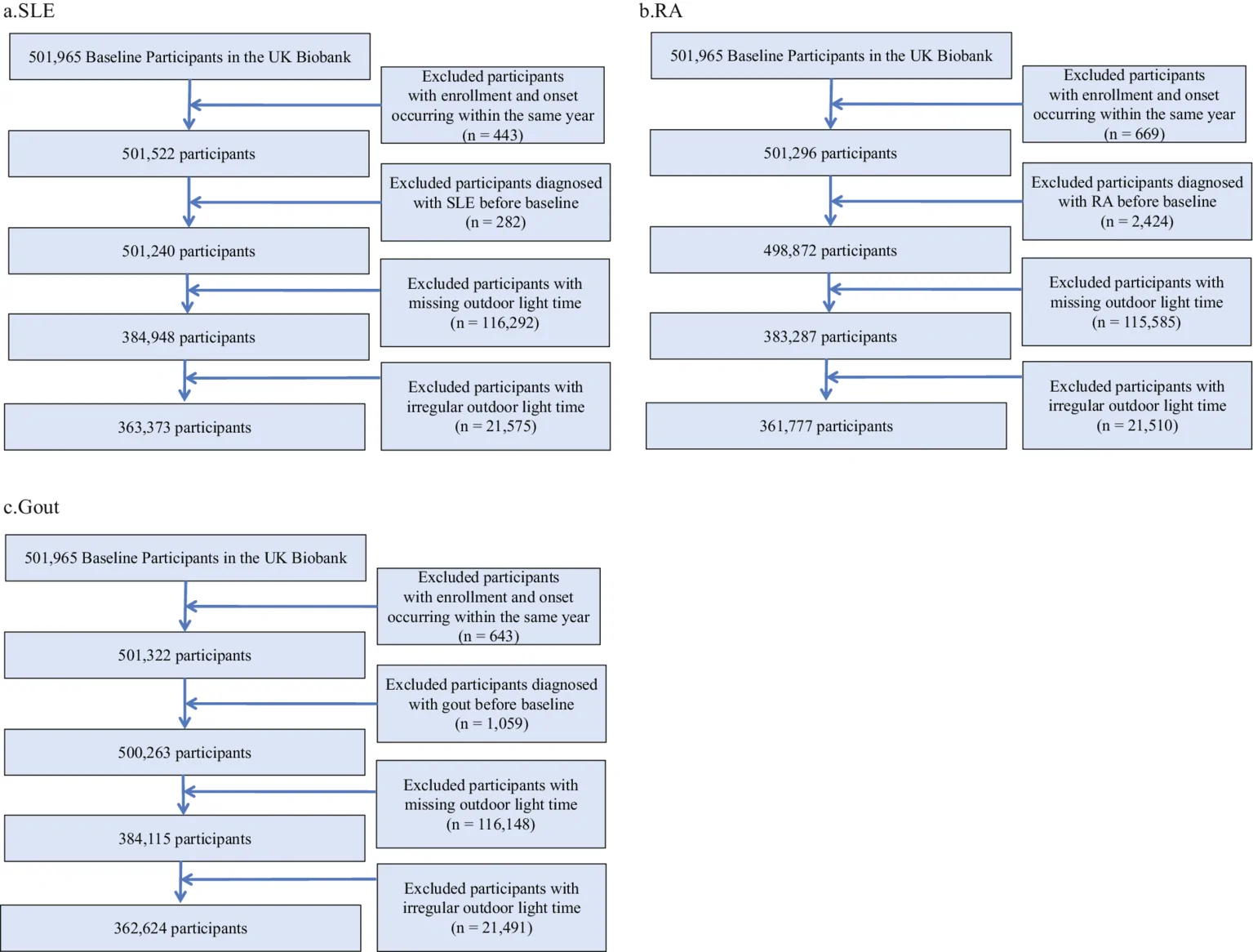
Participant selection flow for outdoor light exposure and rheumatic diseases. **(a)** Systemic lupus erythematosus (SLE); **(b)** rheumatoid arthritis (RA); **(c)** gout.

### Statistical analysis

Baseline characteristics by sunlight exposure levels were summarized as means (SD) for continuous variables and counts (percentages) for categorical variables, with group differences tested using analysis of variance or χ^2^ tests as appropriate. Sunlight exposure duration was trichotomized at the median (< 2.5, 2.5 and > 2.5 h/day). Multivariate Cox proportional hazard regression models were used to examine the associations of sunlight exposure with systemic lupus erythematosus, rheumatoid arthritis and gout. The estimates using hazard ratios (HRs) and the corresponding 95% confidence intervals (95% CIs) compared with regular sunlight exposure as 2.5 h (median values). To assess the potential impact of unmeasured confounding, we calculated E-values, which quantify the minimum strength of association that an unmeasured confounder would need to fully explain away the observed exposure–outcome association. Potential non-linear associations between sunlight exposure and risk of diseases were evaluated using restricted cubic splines with three knots to assess potential nonlinearity based on minimum Akaike information criterion. Subgroup analyses were conducted to examine the consistency of the associations across background or lifestyle strata. Participants were stratified by age (< 65 years and ≥ 65 years), sex (male and female), BMI (< 24.0, 24.0 ~ 27.9, and ≥ 28.0), smoking (yes and no), alcohol consumption (yes and no), physical activity (yes and no), and sleep duration (≤ 7, > 7), and interaction terms between the exposure and subgroup variables were included in the models to formally test for effect modification. Stratified hazard ratios and 95% confidence intervals were estimated within each subgroup. Statistical significance for interaction was assessed using the likelihood ratio test, comparing models with and without interaction terms.

Certain sensitivity analyses were conducted. First, we also conducted Cox models to investigate the associations of sunlight exposure with rheumatic diseases during summer (median: 4 h) or winter (median: 2 h) season, respectively. Second, their associations were examined using inverse probability weighting. Inverse probability weighting was used to address potential baseline confounding. Stabilized weights were derived from logistic models of exposure on covariates, creating a pseudo-population with balanced confounders across exposure groups. Weighted analyses provided effect estimates under the assumption of no unmeasured confounding. Third, as a novel causal inference approach, Cox models with inverse probability weighting were used to estimate the causal effect of average sunlight exposure on disease onset using average treatment effect (ATE) and average treatment effect on the treated (ATT). Weighted Cox models provided marginal effect estimates with hazard ratios and 95% confidence intervals. Fourth, we excluded non-white population, or participants with self-reported poor health or missing covariates. Fifth, we examined the association between average outdoor activity duration and serum 25-hydroxyvitamin D [25(OH)D] concentrations. We further assessed whether the association between average outdoor activity duration and rheumatic disease risk differed across strata of serum 25(OH)D concentrations. In addition, we compared the incidence proportions of SLE, RA, and gout between White and non-White participants.

## Results

A total of 363,873 participants with available sunlight exposure data were included in the analyses. Of these, 138,171 had outdoor sunlight exposure of less than 2.5 h per day, 46,257 had approximately 2.5 h per day, and 179,445 had more than 2.5 h per day. For age distribution, 288,557 participants (79.3%) were younger than 65 years and 75,316 (20.7%) were 65 years or older. For sex distribution, 193,627 participants (53.2%) were female and 170,246 (46.8%) were male. Most participants were White (346,126 [95.1%]). Compared with participants reporting shorter outdoor time, those reporting more than 2.5 h/day were older, more likely to be male, and less likely to have a college education. We found most participants had a white skin color (272,220 in all population) and used sun exposure protection (326,601 in all population) (Table 1).

**Table 1:** Baseline characteristics from 363,873 participants.

Variable name	Category levels	Time spent in outdoor light (average)				*p*
		Overall	< 2.5 h	2.5 h	> 2.5 h	
		*N* = 363,873	*N* = 138,171	*N* = 46,257	*N* = 179,445	
Age, years, *N* (%)	<65	288,557 (79.30)	119,614 (86.57)	37,074 (80.15)	131,869 (73.49)	<0.001
	≥ 65	75,316 (20.70)	18,557 (13.43)	9,183 (19.85)	47,576 (26.51)	
Sex, *N* (%)	Female	193,627 (53.21)	78,031 (56.47)	26,656 (57.63)	88,940 (49.56)	<0.001
	Male	170,246 (46.79)	60,140 (43.53)	19,601 (42.37)	90,505 (50.44)	
Ethnicity, *N* (%)	Non-White	16,649 (4.58)	6,731 (4.87)	1922 (4.16)	7,996 (4.46)	<0.001
	White	346,126 (95.12)	131,047 (94.84)	44,184 (95.52)	170,895 (95.24)	
	Missing	1,098 (0.30)	393 (0.28)	151 (0.33)	554 (0.31)	
Educational levels, *N* (%)	College	116,672 (32.06)	56,886 (41.17)	15,817 (34.19)	43,969 (24.50)	<0.001
	Other levels	182,828 (50.25)	66,357 (48.03)	23,556 (50.92)	92,915 (51.78)	
	Unknown	64,373 (17.69)	14,928 (10.80)	6,884 (14.88)	42,561 (23.72)	
Body mass index, kg/m^2^, *N* (%)	< 24	120,458 (33.10)	49,435 (35.78)	16,026 (34.65)	54,997 (30.65)	<0.001
	24–27.9	156,454 (43.00)	57,101 (41.33)	19,553 (42.27)	79,800 (44.47)	
	≥ 28	85,239 (23.43)	30,983 (22.42)	10,469 (22.63)	43,787 (24.40)	
	Missing	1722 (0.47)	652 (0.47)	209 (0.45)	861 (0.48)	
Physical activity, *N* (%)	Yes	231,576 (63.64)	78,818 (57.04)	28,762 (62.18)	123,996 (69.10)	<0.001
	No	127,198 (34.96)	58,023 (41.99)	16,943 (36.63)	52,232 (29.11)	
	Missing	5,099 (1.40)	1,330 (0.96)	552 (1.19)	3,217 (1.79)	
Drinking status, *N* (%)	Yes	349,019 (95.92)	132,438 (95.85)	44,558 (96.33)	172,023 (95.86)	<0.001
	No	14,553 (4.00)	5,630 (4.07)	1,663 (3.60)	7,260 (4.05)	
	Missing	301 (0.08)	103 (0.07)	36 (0.08)	162 (0.09)	
Smoking status, *N* (%)	Yes	166,492 (45.76)	57,847 (41.87)	20,674 (44.69)	87,971 (49.02)	<0.001
	No	196,208 (53.92)	79,997 (57.90)	25,465 (55.05)	90,746 (50.57)	
	Missing	1,173 (0.32)	327 (0.24)	118 (0.26)	728 (0.41)	
Household income, *£*, *N* (%)	< 31,000	154,560 (42.48)	47,548 (34.41)	18,803 (40.65)	88,209 (49.16)	<0.001
	≥ 31,000	160,445 (44.09)	75,923 (54.95)	21,489 (46.46)	63,033 (35.13)	
	Missing	48,868 (13.43)	14,700 (10.64)	5,965 (12.90)	28,203 (15.72)	
Dieting score, *N* (%)	0–3	252,213 (69.31)	98,034 (70.95)	32,086 (69.36)	122,093 (68.04)	<0.001
	4–7	111,646 (30.68)	40,133 (29.05)	14,168 (30.63)	57,345 (31.96)	
	Missing	14 (0.00)	4 (0.00)	3 (0.01)	7 (0.00)	
Sleep duration, hours, *N* (%)	≤7	226,762 (62.32)	91,448 (66.18)	28,942 (62.57)	106,372 (59.28)	<0.001
	>7	136,117 (37.41)	46,421 (33.60)	17,208 (37.20)	72,488 (40.40)	
	Missing	994 (0.27)	302 (0.22)	107 (0.23)	585 (0.33)	
Ambient PM_2.5_ levels, μg/m^3^, mean (SD)		9.98 (1.01)	10.01 (1.02)	9.95 (0.99)	9.95 (1.01)	<0.001
Skin color, *N* (%)	Other	87,602 (24.07)	30,713 (22.23)	11,030 (23.85)	45,859 (25.56)	<0.001
	White	272,220 (74.81)	106,229 (76.88)	34,723 (75.07)	131,268 (73.15)	
	Missing	4,051 (1.11)	1,229 (0.89)	504 (1.09)	2,318 (1.29)	
Use of sun exposure protection, *N* (%)	No	35,201 (9.67)	12,728 (9.21)	3,835 (8.29)	18,638 (10.39)	<0.001
	Yes	326,601 (89.76)	124,375 (90.02)	42,226 (91.29)	160,000 (89.16)	
	Missing	2071 (0.57)	1,068 (0.77)	196 (0.42)	807 (0.45)	

During the follow-up between 2006 and 2022, a total of 335 incident cases of systemic lupus erythematosus, 5,591 cases of rheumatoid arthritis, and 7,485 cases of gout were documented (Table 2). Compared to sunlight exposure at 2.5 h, the HRs and 95% CIs under 2.5 h were 1.71 (95% CIs: 1.13, 2.59) for systemic lupus erythematosus, 1.05 (95% CIs: 0.96, 1.14) for rheumatoid arthritis and 0.96 (95% CIs: 0.88, 1.04) for gout; the HRs and 95% CIs above 2.5 h were 1.75 (95% CIs: 1.17, 2.63) for systemic lupus erythematosus, 1.15 (95% CIs:1.06, 1.26) for rheumatoid arthritis and 1.09 (95% CIs: 1.02, 1.18) for gout. The observed E-values ranged from 1.25 to 2.81 for participants with less than 2.5 h of outdoor sunlight exposure and from 1.40 to 2.89 for those with more than 2.5 h, indicating that only unmeasured confounders of substantial strength could fully explain these associations.

**Table 2:** Associations of sunlight exposure with systemic lupus erythematosus, rheumatoid arthritis, and gout in the UK.

Outdoor duration (hours)	Cases/Person-years	HR (95% CI)	*p* value	E value
Systemic lupus erythematosus, SLE				
2.5	27/616357	1	NA	NA
< 2.5	133/1847551	**1.71 (1.13–2.59)**	**0.011**	**2.81**
> 2.5	175/2370470	**1.75 (1.17–2.63)**	**0.007**	**2.89**
Rheumatoid arthritis, RA				
2.5	645/610485	1	NA	NA
< 2.5	1836/1831941	1.05 (0.96–1.14)	0.333	**1.28**
> 2.5	3110/2343037	**1.15 (1.06–1.26)**	**0.001**	**1.57**
Gout				
2.5	827/611821	1	NA	NA
< 2.5	2194/1836420	0.96 (0.88–1.04)	0.304	**1.25**
> 2.5	4464/2345562	**1.09 (1.02–1.18)**	**0.018**	**1.40**

Models were adjusted for age (< 65 years and ≥ 65 years), sex (Male and Female), ethnicity (White and Non-white), educational levels (College and Other levels), Body mass index (BMI) (kg/m^2^) (< 24, 24 ~ 27.9, ≥ 28), alcohol consumption (Yes and No), smoking status (Yes and No), household income (< £ 31,000 and ≥ £ 31,000), physical activity (Yes and No), dieting score (ranged from 0–7), sleep duration (≤ 7 h and > 7 h), ambient PM2.5 levels (μg/m^3^), skin color (White and Other), use of sun exposure protection (Yes and No). Bold values indicate statistically significant associations (*p* < 0.05).

We used restricted cubic splines to fit the nonlinearity of sunlight exposure time and rheumatic diseases (Figure 2). No significant nonlinearity was detected for SLE, RA, or gout (*p* for nonlinearity > 0.05).

**Figure 2:**
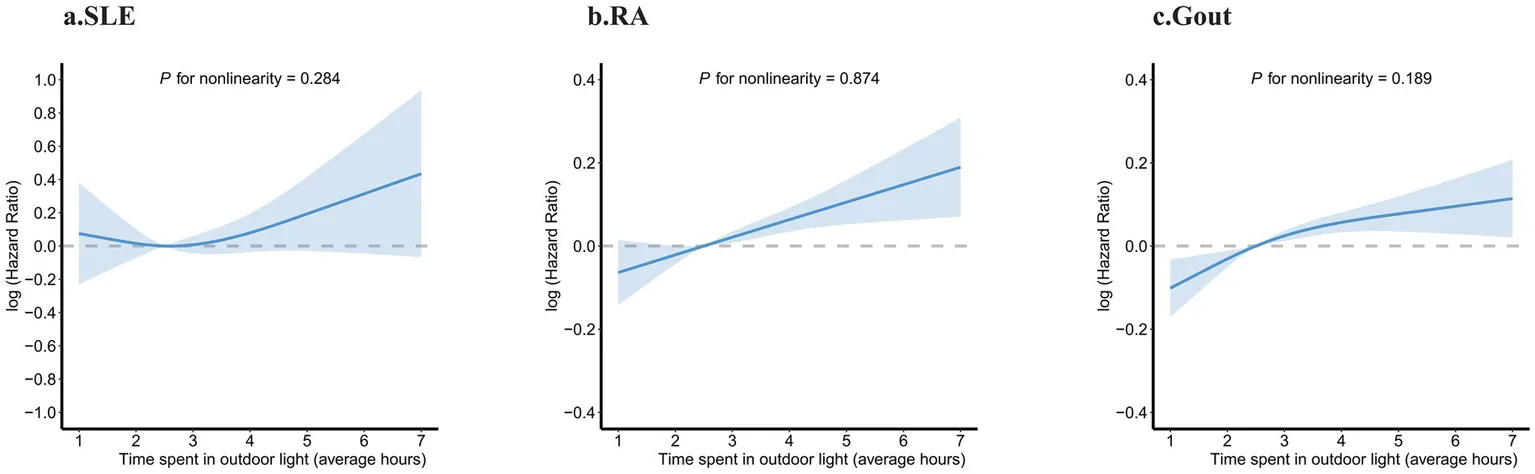
Restricted cubic spline analysis of outdoor light exposure and rheumatic disease risk. **(a)** Systemic lupus erythematosus (SLE); **(b)** rheumatoid arthritis (RA); **(c)** gout. Restricted cubic spline regression was used. Models were adjusted for age (< 65 years and ≥ 65 years), sex (Male and Female), ethnicity (White and Non-white), educational levels (College and Other levels), body mass index (BMI) (kg/m^2^) (< 24, 24 ~ 27.9, ≥ 28), alcohol consumption (Yes and No), smoking status (Yes and No), household income (< £ 31,000 and ≥ £ 31,000), physical activity (Yes and No), dieting score (ranged from 0–7), sleep duration (≤ 7 h and > 7 h), ambient PM_2.5_ levels (μg/m^3^), skin color (White and Other), use of sun exposure protection (Yes and No).

In the subgroup analyses, several nominal interactions were observed before correction (smoking in SLE, *p* for interaction = 0.025, Table 3; age and BMI in RA, *p* = 0.043 and *p* < 0.01, Table 4). However, after Bonferroni correction these were no longer significant and should be regarded as exploratory. Only the interaction for age in gout remained significant after correction (adjusted *p* = 0.014), with a higher risk of gout associated with longer sunlight exposure (> 2.5 h) among participants aged ≥ 65 years (Table 5).

**Table 3:** Subgroup analysis of sunlight exposure and systemic lupus erythematosus.

Systemic lupus erythematosus, SLE	Cases	Time spent in outdoor light (average)					
		2.5 h	< 2.5 h		> 2.5 h		*p* for interaction (Adjusted *p* value)
		Reference	HR	*p*	HR	*p*	
Age, years							0.133 (0.931)
< 65	267	1	1.86 (1.14–3.03)	0.013	2.09 (1.29–3.38)	0.003	
≥ 65	68	1	1.46 (0.65–3.27)	0.357	1.00 (0.46–2.15)	0.991	
Sex							0.236 (1.000)
Male	60	1	1.87 (0.54–6.44)	0.321	2.66 (0.82–8.66)	0.103	
Female	275	1	**1.69 (1.09–2.62)**	**0.020**	**1.60 (1.04–2.48)**	**0.034**	
Body mass index (kg/m^2^)							0.578 (1.000)
<24	128	1	**2.17 (1.03–4.58)**	**0.042**	**2.57 (1.23–5.36)**	**0.012**	
24.0–27.9	117	1	1.43 (0.74–2.79)	0.287	1.45 (0.76–2.75)	0.262	
≥ 28.0	90	1	1.66 (0.78–3.57)	0.191	1.38 (0.65–2.95)	0.404	
Smoking							0.025 (0.175)
Yes	171	1	1.07 (0.63–1.83)	0.807	1.41 (0.85–2.33)	0.183	
No	164	1	**2.98 (1.49–5.94)**	**0.002**	**2.40 (1.20–4.80)**	**0.013**	
Drinking							0.941 (1.000)
Yes	316	1	**1.69 (1.11–2.58)**	**0.015**	**1.72 (1.13–2.60)**	**0.011**	
No	19	1	2.49 (0.31–20.01)	0.39	2.50 (0.32–19.62)	0.384	
Physical activity							0.922 (1.000)
Yes	205	1	1.64 (0.96–2.78)	0.069	1.65 (0.99–2.74)	0.056	
No	130	1	1.85 (0.94–3.61)	0.073	1.93 (0.99–3.78)	0.055	
Sleep duration, hours							0.483 (1.000)
≤7	206	1	1.69 (0.99–2.89)	0.056	**1.93 (1.14–3.27)**	**0.014**	
> 7	129	1	1.78 (0.93–3.41)	0.084	1.50 (0.79–2.86)	0.213	

Models were adjusted for age (< 65 years and ≥ 65 years), sex (Male and Female), ethnicity (White and Non-white), educational levels (College and Other levels), Body mass index (BMI) (kg/m^2^) (< 24, 24 ~ 27.9, ≥ 28), alcohol consumption (Yes and No), smoking status (Yes and No), household income (< £ 31,000 and ≥ £ 31,000), physical activity (Yes and No), dieting score (ranged from 0–7), sleep duration (≤ 7 h and > 7 h), ambient PM_2.5_ levels (μg/m^3^), skin color (White and Other), use of sun exposure protection (Yes and No) except for the stratifying variable. Bold values indicate statistically significant associations (*p* < 0.05).

**Table 4:** Subgroup analysis of sunlight exposure and rheumatoid arthritis.

Rheumatoid arthritis, RA	Cases	Time spent in outdoor light (average)					*p* for interaction
		2.5 h	< 2.5 h		> 2.5 h		
		Reference	HR	*p* value	HR	*p* value	(Adjusted *p* value)
Age, years							0.043 (0.301)
< 65	3,852	1	**1.07 (0.96–1.19)**	**0.236**	**1.22 (1.10–1.35)**	**< 0.001**	
≥ 65	1739	1	1.02 (0.87–1.21)	0.773	1.03 (0.88–1.19)	0.738	
Sex							0.327 (1.000)
Male	1888	1	1.05 (0.89–1.25)	0.566	1.18 (1.01–1.38)	0.038	
Female	3,703	1	1.04 (0.94–1.16)	0.445	**1.13 (1.02–1.26)**	**0.016**	
Body mass index (kg/m^2^)							0.008 (0.056)
<24	1,504	1	0.85 (0.72–1.00)	0.055	1.03 (0.88–1.20)	0.742	
24.0–27.9	2,302	1	**1.18 (1.02–1.37)**	**0.024**	**1.26 (1.10–1.45)**	**0.001**	
≥ 28.0	1785	1	1.08 (0.92–1.26)	0.369	1.15 (0.99–1.34)	0.076	
Smoking							0.224 (1.000)
Yes	3,050	1	1.10 (0.97–1.25)	0.147	**1.22 (1.08–1.37)**	0.001	
No	2,541	1	0.99 (0.87–1.13)	0.904	1.08 (0.96–1.23)	0.203	
Drinking							0.347 (1.000)
Yes	5,236	1	1.04 (0.95–1.14)	0.385	**1.16 (1.06–1.27)**	**0.001**	
No	355	1	1.10 (0.77–1.57)	0.619	1.05 (0.74–1.49)	0.785	
Physical activity							0.212 (1.000)
Yes	3,466	1	1.01 (0.89–1.13)	0.927	**1.16 (1.04–1.30)**	**0.007**	
No	2,125	1	1.10 (0.95–1.26)	0.201	1.13 (0.99–1.30)	0.070	
Sleep duration, hours							0.133 (0.931)
≤7	3,459	1	0.98 (0.87–1.09)	0.700	1.10 (0.99–1.23)	0.072	
> 7	2,132	1	**1.18 (1.01–1.38)**	**0.032**	**1.25 (1.08–1.44)**	**0.002**	

Models were adjusted for age (< 65 years and ≥ 65 years), sex (Male and Female), ethnicity (White and Non-white), educational levels (College and Other levels), Body mass index (BMI) (kg/m^2^) (< 24, 24 ~ 27.9, ≥ 28), alcohol consumption (Yes and No), smoking status (Yes and No), household income (< £ 31,000 and ≥ £ 31,000), physical activity (Yes and No), dieting score (ranged from 0–7), sleep duration (≤ 7 h and > 7 h), ambient PM_2.5_ levels (μg/m^3^), skin color (White and Other), use of sun exposure protection (Yes and No) except for the stratifying variable. Bold values indicate statistically significant associations (*p* < 0.05).

**Table 5:** Subgroup analysis of sunlight exposure and gout.

Gout	Cases	Time spent in outdoor light (average)					*p* for interaction (Adjusted *p* value)
		2.5 h	< 2.5 h		> 2.5 h		
		Reference	HR	*p* value	HR	*p* value	
Age, y							0.002 (0.014)
< 65	4,760	1	0.98 (0.85–1.12)	0.731	1.03 (0.91–1.17)	0.591	
≥ 65	2,725	1	0.97 (0.88–1.07)	0.511	**1.14 (1.04–1.25)**	**0.007**	
Sex							0.055 (0.385)
Male	6,264	1	0.99 (0.91–1.09)	0.855	**1.14 (1.04–1.23)**	**0.003**	
Female	1,221	1	**0.83 (0.70–1.00)**	**0.045**	0.97 (0.82–1.14)	0.688	
Body mass index							0.096 (0.672)
<24	792	1	0.90 (0.71–1.14)	0.393	1.02 (0.82–1.28)	0.830	
24.0–27.9	3,292	1	0.95 (0.84–1.08)	0.465	**1.15 (1.03–1.29)**	**0.017**	
≥ 28.0	3,401	1	0.98 (0.87–1.10)	0.683	1.06 (0.95–1.18)	0.309	
Smoking							0.904 (1.000)
Yes	4,466	1	0.96 (0.86–1.06)	0.410	1.10 (1.00–1.21)	0.060	
No	3,019	1	0.96 (0.85–1.09)	0.543	1.09 (0.97–1.22)	0.161	
Drinking							0.959 (1.000)
Yes	7,287	1	0.96 (0.88–1.04)	0.286	**1.09 (1.01–1.18)**	**0.026**	
No	198	1	1.05 (0.63–1.75)	0.863	1.28 (0.79–2.09)	0.316	
Physical activity							0.566 (1.000)
Yes	4,608	1	0.99 (0.89–1.11)	0.879	**1.13 (1.03–1.25)**	**0.012**	
No	2,877	1	0.92 (0.81–1.03)	0.155	1.04 (0.93–1.17)	0.499	
Sleep duration, h							0.081 (0.567)
≤7	4,462	1	1.00 (0.90–1.11)	0.980	**1.16 (1.05–1.28)**	**0.003**	
> 7	3,023	1	0.91 (0.80–1.03)	0.124	1.00 (0.89–1.13)	0.953	

Models were adjusted for age (< 65 years and ≥ 65 years), sex (Male and Female), ethnicity (White and Non-white), educational levels (College and Other levels), Body mass index (BMI) (kg/m^2^) (< 24, 24 ~ 27.9, ≥ 28), alcohol consumption (Yes and No), smoking status (Yes and No), household income (< £ 31,000 and ≥ £ 31,000), physical activity (Yes and No), dieting score (ranged from 0–7), sleep duration (≤ 7 h and > 7 h), ambient PM_2.5_ levels (μg/m^3^), skin color (White and Other), use of sun exposure protection (Yes and No) except for the stratifying variable. Bold values indicate statistically significant associations (*p* < 0.05).

A series of sensitivity analyses were conducted. We observed the associations of sunlight exposure with systemic lupus erythematosus and gout in summer (Supplementary Table S2), as well as with gout in winter (Supplementary Table S3). The associations of sunlight exposure with systemic lupus erythematosus, rheumatoid arthritis, and gout remained consistent after using inverse probability weighting (Supplementary Table S4), only including white population (Supplementary Table S5), excluding poor health (Supplementary Table S6) and missing covariates (Supplementary Table S7). Compared with 2.5 h of outdoor exposure, < 2.5 h was associated with lower odds, while >2.5 h was associated with higher odds of above-mean serum 25(OH)D concentrations (Supplementary Table S8). In analyses stratified by serum 25(OH)D levels, we observed a significant interaction for SLE after Bonferroni correction, although the stratum-specific estimates were imprecise. No significant interaction was observed for RA or gout after correction (Supplementary Table S9). The incidence proportions of SLE and RA were numerically higher among non-White participants than among White participants, whereas the incidence proportion of gout was similar between the two groups (Supplementary Table S10).

## Discussion

In this large prospective cohort, sunlight exposure showed distinct associations with incident SLE, RA, and gout. Both shorter and longer outdoor time were associated with higher SLE risk relative to 2.5 h/day, whereas longer outdoor time was associated with modestly increased risks of RA and gout. These differences suggest that sunlight-related exposures may interact with disease-specific pathogenic backgrounds, although the modest effect sizes for RA and gout and the observational nature of the study warrant cautious interpretation.

Among rheumatic diseases, SLE represents the condition most strongly associated with sunlight exposure (6). Photosensitivity is a well-recognized feature of SLE, and ultraviolet radiation has been implicated in disease pathogenesis through keratinocyte apoptosis, autoantigen release, and type I interferon activation (8, 10). Although ultraviolet exposure has been widely implicated in SLE susceptibility, previous studies have mainly focused on geographic variation, environmental associations, or disease manifestations among patients with established SLE (3, 14, 15). However, prospective evidence linking habitual sunlight exposure to incident SLE remains limited. In our study, both shorter and longer outdoor time were associated with higher SLE risk, suggesting that the effects of sunlight exposure may vary according to exposure patterns and host susceptibility. This finding may reflect the context-dependent immunological effects of ultraviolet radiation.

Previous studies have suggested that the relationship between sunlight exposure and RA risk is less well established and more heterogeneous than that observed in SLE. Prospective evidence has indicated a potential inverse association between ambient UV-B exposure and RA risk, which may be partly attributable to vitamin D-mediated immunoregulation (16, 17). However, this association has not been consistently reproduced across populations, and population-based analyses have not identified a clear relationship between sunlight exposure and ACPA positivity (18). In our study, longer outdoor time was associated with a modestly increased risk of incident RA, suggesting that the biological impact of sunlight exposure on RA development may reflect a complex interplay among vitamin D-related pathways, UV-mediated immune responses, and other environmental determinants.

Compared with SLE and RA, the role of environmental factors in gout has received relatively less attention, with previous studies mainly focusing on metabolic and toxic exposures (19–21). Gout is characterized by urate metabolism dysregulation and monosodium urate crystal-induced innate immune activation (22). Although environmental exposures such as lead have been linked to gout risk (23, 24), the potential contribution of sunlight exposure to gout development remains poorly understood. In our study, longer outdoor time was associated with increased gout risk, particularly among older participants, suggesting that age-related metabolic susceptibility may influence the relationship between sunlight exposure and gout risk.

These cut-points also warrant external context. The median of 2.5 h/day (4 h/day in summer and 2 h/day in winter) is comparable with other analyses of the same UK Biobank item (25) and is not low by general-population standards, since US respondents reported spending only about 7.6% of the day, roughly 1.8 h, outdoors (26). Time outdoors is nevertheless not equivalent to ultraviolet dose: the United Kingdom lies between about 50° N and 58° N, and vitamin D-effective ultraviolet-B doses differ up to five-fold across Europe (27), so 2.5 h here delivers considerably less ultraviolet radiation than the same duration at lower latitudes. Disease burden varies geographically in parallel, with SLE reported more frequently in high-income countries (28) and gout driven largely by aging and metabolic factors (29). Consistent with these being distinct exposure metrics, studies of ambient ultraviolet-B reported no association with SLE (15) and an inverse association with RA (16). Our median-based cut-points are therefore population-specific and should not be transferred to higher-ultraviolet settings.

The methodological strengths of this study include its large-scale cohort spanning 2006 to 2022 and the use of multiple statistical approaches. Furthermore, this research provides a systematic comparison of three distinct rheumatic diseases within a unified cohort, offering unique insights into disease-specific variations. Several methodological limitations warrant consideration. The most important is the reliance on self-reported sunlight exposure, which is a major limitation in its own right and not merely a matter of unresolved wavelengths. Recall of a “typical” summer and winter day at a single baseline visit is subject to recall error, rounding and social-desirability bias, and cannot capture time of day, day-to-day variability, or shade and clothing use; self-reported exposure agrees only fair-to-moderately with personal ultraviolet dosimetry, with correlations of approximately 0.3 to 0.5 (30, 31). Non-differential error of this kind would attenuate hazard ratios toward the null and may contribute to the modest estimates for RA and gout (32), whereas reporting related to prodromal symptoms could bias estimates in either direction. UK Biobank also collected no items on occupational or recreational sun exposure, and neither validated instruments such as the Sun Exposure and Protection Index (33) nor occupational job-exposure matrices (34) were available to us; future cohorts should embed these and, where feasible, calibrate them against personal dosimetry. A second major limitation is that sun protection was ascertained with a single baseline question and modeled as a binary variable, whereas it is a time-varying habit that is difficult to quantify. A yes/no indicator distinguishes neither the modality of protection nor its frequency or adequacy of application; sun protection factors are determined at an application thickness of 2 mg/cm^2^, whereas real-world application is typically only 0.4 to 1.0 mg/cm^2^, so the protection achieved is well below the labeled value (35). Protective behavior may also change once symptoms appear. Residual confounding by sun protection therefore probably persists, and our E-values (1.25 to 2.89), while informative, do not remove this concern. The predominantly White UK population and distinctive spectral characteristics at high latitudes limit generalizability to other geographic and ethnic contexts. Additionally, despite extensive covariate adjustment, residual confounding from unmeasured factors such as genetic susceptibility and skin barrier function variations may persist. Our findings carry important implications for public health practice.

Despite these limitations, our study suggests that sunlight exposure should not be viewed simply as universally harmful or beneficial. Rather, its health effects may depend on the underlying disease context. In SLE, where photosensitivity is a well-recognized clinical feature, our findings support the continued emphasis on moderate sunlight exposure and appropriate sun protection. Short outdoor activities may be considered in the early morning or late afternoon, when UV intensity is relatively low and red or near-infrared light is more abundant. In contrast, for patients with RA or gout, the modest associations we observed do not support excessive restriction of outdoor activity. A more balanced approach, combining regular outdoor activity with reasonable sun protection, may be more appropriate. Future studies using objective light-monitoring tools could help distinguish the effects of different wavelengths and exposure durations, and could provide a stronger basis for individualized sunlight exposure guidance in patients with rheumatic diseases.

## Conclusion

In this large prospective cohort, sunlight exposure was associated with the risks of SLE, RA, and gout in a disease-specific manner. These findings suggest that sunlight exposure should not be considered a uniform environmental factor across rheumatic diseases, but rather one whose health implications may depend on the underlying disease context. Further studies using objective exposure assessment and more diverse populations are needed to clarify these associations and to support more tailored guidance on sunlight exposure and sun protection.

## Data Availability

The data used in the present study are available from the UK Biobank with restrictions applied. Access to the UK Biobank data can be requested by means of a standard protocol (https://www.ukbiobank.ac.uk/register-apply/). Requests to access these datasets should be directed to https://www.ukbiobank.ac.uk/register-apply/.
