# Supplementary material for: Association between sunlight exposure and risk of rheumatic diseases: a prospective cohort study in the UK
Source: Front Public Health. 2026 Sep 14;14:1873382. doi: 10.3389/fpubh.2026.1873382 (PMC13617017; doi:10.3389/fpubh.2026.1873382)
Supplement: Supplementary file 1 [file Table_1.docx]

**Supplementary Files**

**Sunlight Exposure and Incident Rheumatic Diseases in the UK**

**Table S1.** Disease definition based on ICD-10 codes.

**Table S2.** Associations of outdoor sunlight exposure in summer with the risk of incident SLE, RA and gout among UK participants.

**Table S3.** Associations of outdoor sunlight exposure in winter with the risk of incident SLE, RA and gout among UK participants.

**Table S4.** Associations of average sunlight exposure with the risk of incident SLE, RA and gout using inverse probability weighting.

**Table S5.** Associations of average sunlight exposure with the risk of incident SLE, RA and gout (excluding non-white population).

**Table S6.** Associations of average sunlight exposure with the risk of incident SLE, RA and gout (excluding participants with poor self-reported health).

**Table S7.** Associations of average sunlight exposure with the risk of incident SLE, RA and gout (excluding participants with missing values).

**Table S8.** Associations between average sunlight exposure and serum 25-hydroxyvitamin D concentrations in SLE, RA and gout cohort.

**Table S9.** Associations of average sunlight exposure with the risk of SLE, RA and gout stratified by serum 25-hydroxyvitamin D levels.

**Table S10**. Incidence proportions of SLE, RA, and gout according to ethnicity in the UK Biobank cohort.

**Table S1.** Disease definition based on ICD 10 code.

| **ICD 10 codes** | **Diseases** |
| --- | --- |
| M320; M321;M328;M329 | Systemic lupus erythematosus, SLE |
| M050; M051; M052; M053; M058; M059; M060 ; M061; M062; M063; M064; M068; M069; M05; M06 | Rheumatoid Arthritis, RA |
| M100','M1000', 'M1001', 'M1002', 'M1003', 'M1004', 'M1006', 'M1007', 'M1008', 'M1009', 'M101', 'M102','M1020', 'M1024','M1025', 'M1027', 'M1029','M103', 'M1030','M1036','M1037', 'M1039', 'M104', 'M1040', 'M1045','M1046', 'M1049', 'M109', 'M1090', 'M1091', 'M1092', 'M1093', 'M1094', 'M1095', 'M1096', 'M1097', 'M1098', 'M1099' | Gout |

**Table S2.** Associations of outdoor light time in summer with the risk of incident SLE, RA and gout among UK participants.

| **Outdoor duration (hours)** | **Cases** | **HR (95% CI)** | ***P* value** | **E value** |
| --- | --- | --- | --- | --- |
|  |  |  |  |  |
| **Systemic lupus erythematosus, SLE** |  |  |  |  |
| 4 | 54 | 1 | NA | NA |
| < 4 | 160 | 1.29 (0.91–1.84) | 0.148 | 1.90 |
| > 4 | 125 | 1.21 (0.83–1.75) | 0.322 | 1.71 |
| **Rheumatoid Arthritis, RA** |  |  |  |  |
| 4 | 1065 | 1 | NA | NA |
| < 4 | 2401 | 1.02 (0.95–1.10) | 0.593 | 1.16 |
| > 4 | 2144 | **1.11 (1.03–1.20)** | **0.008** | 1.46 |
| **Gout** |  |  |  |  |
| 4 | 1421 | 1 | NA | NA |
| < 4 | 2858 | **0.93 (0.87–1.00)** | **0.041** | 1.36 |
| > 4 | 3243 | 1.06 (0.99–1.13) | 0.117 | 1.31 |

Models were adjusted for age (< 65 years and ≥ 65 years), sex (Male and Female), ethnicity (White and Non-white), educational levels (College and Other levels), Body mass index (BMI) (kg/m^2^) (< 24, 24~27.9, ≥ 28), alcohol consumption (Yes and No), smoking status (Yes and No), household income (< £ 31,000 and ≥ £ 31,000), physical activity (Yes and No), dieting score (ranged from 0-7), sleep duration (≤ 7 hours and > 7 hours), ambient PM_2.5_ levels (μg/m^3^), skin color (White and Other), use of sun exposure protection (Yes and No).

**Table S3.** Associations of outdoor light time in winter with the risk of incident SLE, RA and gout among UK participants.

| **Outdoor duration (hours)** | **Cases** | **HR (95% CI)** | ***P* value** | **E value** |
| --- | --- | --- | --- | --- |
|  |  |  |  |  |
| **Systemic lupus erythematosus, SLE** |  |  |  |  |
| 2 | 112 | 1 | NA | NA |
| < 2 | 126 | 0.84 (0.63–1.13) | 0.254 | 1.67 |
| > 2 | 81 | 0.89 (0.63–1.25) | 0.500 | 1.5 |
| **Rheumatoid Arthritis, RA** |  |  |  |  |
| 2 | 1708 | 1 | NA | NA |
| < 2 | 2101 | 0.98 (0.91–1.04) | 0.477 | 1.16 |
| > 2 | 1590 | 1.02 (0.95–1.10) | 0.527 | 1.16 |
| **Gout** |  |  |  |  |
| 2 | 2214 | 1 | NA | NA |
| < 2 | 2552 | 0.96 (0.90–1.02) | 0.218 | 1.25 |
| > 2 | 2624 | **1.07 (1.01–1.14)** | **0.032** | 1.34 |

Models were adjusted for age (< 65 years and ≥ 65 years), sex (Male and Female), ethnicity (White and Non-white), educational levels (College and Other levels), Body mass index (BMI) (kg/m^2^) (< 24, 24~27.9, ≥ 28), alcohol consumption (Yes and No), smoking status (Yes and No), household income (< £ 31,000 and ≥ £ 31,000), physical activity (Yes and No), dieting score (ranged from 0-7), sleep duration (≤ 7 hours and > 7 hours), ambient PM_2.5_ levels (μg/m^3^), skin color (White and Other), use of sun exposure protection (Yes and No).

**Table S4.** Associations of outdoor light time in average with the risk of incident SLE, RA and gout among UK participants based on Cox model using inverse probability weighting.

| **Outdoor duration (hours)** | **Average Treatment Effect (ATE)** | | **Average Treatment effect on the treated (ATT)** | |
| --- | --- | --- | --- | --- |
|  | **HR (95% CI)** | ***P* value** | **HR (95% CI)** | ***P* value** |
|  |  |  |  |  |
| **Systemic lupus erythematosus, SLE** |  |  |  |  |
| <= 2.5 | 1 | NA | 1 | NA |
| > 2.5 | 1.16 (0.93, 1.46) | 0.188 | 1.13 (0.90, 1.43) | 0.290 |
| **Rheumatoid Arthritis, RA** |  |  |  |  |
| < =2.5 | 1 | NA | 1 | NA |
| > 2.5 | **1.10 (1.04, 1.16)** | **< 0.001** | **1.07 (1.01, 1.14)** | **0.015** |
| **Gout** |  |  |  |  |
| <= 2.5 | 1 | NA | 1 | NA |
| > 2.5 | **1.15 (1.10, 1.21)** | **< 0.001** | **1.16 (1.10, 1.22)** | **< 0.001** |

The covariates to be balanced included age (< 65 years and ≥ 65 years), sex (Male and Female), ethnicity (White and Non-white), educational levels (College and Other levels), Body mass index (BMI) (kg/m^2^) (< 24, 24~27.9, ≥ 28), alcohol consumption (Yes and No), smoking status (Yes and No), household income (< £ 31,000 and ≥ £ 31,000), physical activity (Yes and No), dieting score (ranged from 0-7), sleep duration (≤ 7 hours and > 7 hours), ambient PM_2.5_ levels (μg/m^3^), skin color (White and Other), use of sun exposure protection (Yes and No).

**Table S5.** Associations of outdoor light time in average with the risk of incident SLE, RA and gout among UK participants, excluding non-white population.

| **Outdoor duration (hours)** | **HR (95% CI)** | ***P* value** | **E value** |
| --- | --- | --- | --- |
|  |  |  |  |
| **Systemic lupus erythematosus, SLE** |  |  |  |
| 2.5 | 1 | NA | NA |
| < 2.5 | **1.77 (1.14–2.75)** | **0.011** | 2.94 |
| > 2.5 | **1.84 (1.20–2.83)** | **0.006** | 3.08 |
| **Rheumatoid Arthritis, RA** |  |  |  |
| 2.5 | 1 | NA | NA |
| < 2.5 | 1.03 (0.94–1.13) | 0.560 | 1.21 |
| > 2.5 | **1.15 (1.05–1.25)** | **0.002** | 1.57 |
| **Gout** |  |  |  |
| 2.5 | 1 | NA | NA |
| < 2.5 | 0.96 (0.89–1.05) | 0.364 | 1.25 |
| > 2.5 | **1.11 (1.02–1.19)** | **0.011** | 1.46 |

Models were adjusted for age (< 65 years and ≥ 65 years), sex (Male and Female), educational levels (College and Other levels), Body mass index (BMI) (kg/m^2^) (< 24, 24~27.9, ≥ 28), alcohol consumption (Yes and No), smoking status (Yes and No), household income (< £ 31,000 and ≥ £ 31,000), physical activity (Yes and No), dieting score (ranged from 0-7), sleep duration (≤ 7 hours and > 7 hours), ambient PM_2.5_ levels (μg/m^3^), skin color (White and Other), use of sun exposure protection (Yes and No).

**Table S6.** Associations of outdoor light time in average with the risk of incident SLE, RA and gout among UK participants, excluding poor self-report health

| **Outdoor duration (hours)** | **HR (95% CI)** | ***P* value** | **E value** |
| --- | --- | --- | --- |
|  |  |  |  |
| **Systemic lupus erythematosus, SLE** |  |  |  |
| 2.5 | 1 | NA | NA |
| < 2.5 | **1.85 (1.16–2.95)** | **0.010** | 3.10 |
| > 2.5 | **1.88 (1.19–2.98)** | **0.007** | 3.16 |
| **Rheumatoid Arthritis, RA** |  |  |  |
| 2.5 | 1 | NA | NA |
| < 2.5 | 1.03 (0.93–1.13) | 0.591 | 1.21 |
| > 2.5 | **1.14 (1.04–1.25)** | **0.004** | 1.54 |
| **Gout** |  |  |  |
| 2.5 | 1 | NA | NA |
| < 2.5 | 0.94 (0.87–1.03) | 0.178 | 1.32 |
| > 2.5 | **1.10 (1.02–1.19)** | **0.015** | 1.43 |

Models were adjusted for age (< 65 years and ≥ 65 years), sex (Male and Female), ethnicity (White and Non-white), educational levels (College and Other levels), Body mass index (BMI) (kg/m^2^) (< 24, 24~27.9, ≥ 28), alcohol consumption (Yes and No), smoking status (Yes and No), household income (< £ 31,000 and ≥ £ 31,000), physical activity (Yes and No), dieting score (ranged from 0-7), sleep duration (≤ 7 hours and > 7 hours), ambient PM_2.5_ levels (μg/m^3^), skin color (White and Other), use of sun exposure protection (Yes and No).

**Table S7.** Associations of outdoor light time in average with the risk of incident SLE, RA and gout among UK participants, excluding missing values.

| **Outdoor duration (hours)** | **HR (95% CI)** | ***P* value** | **E value** |
| --- | --- | --- | --- |
|  |  |  |  |
| **Systemic lupus erythematosus, SLE** |  |  |  |
| 2.5 | 1 | NA | NA |
| < 2.5 | **1.74 (1.10–2.75)** | **0.018** | 2.87 |
| > 2.5 | 1.53 (0.97–2.41) | 0.070 | 2.43 |
| **Rheumatoid Arthritis, RA** |  |  |  |
| 2.5 | 1 | NA | NA |
| < 2.5 | 1.05 (0.96–1.15) | 0.320 | 1.28 |
| > 2.5 | **1.15 (1.05–1.25)** | **0.002** | 1.56 |
| **Gout** |  |  |  |
| 2.5 | 1 | NA | NA |
| < 2.5 | 0.97 (0.89–1.06) | 0.483 | 1.21 |
| > 2.5 | **1.09 (1.00–1.18)** | **0.048** | 1.4 |

Models were adjusted for age (< 65 years and ≥ 65 years), sex (Male and Female), ethnicity (White and Non-white), educational levels (College and Other levels), Body mass index (BMI) (kg/m^2^) (< 24, 24~27.9, ≥ 28), alcohol consumption (Yes and No), smoking status (Yes and No), household income (< £ 31,000 and ≥ £ 31,000), physical activity (Yes and No), dieting score (ranged from 0-7), sleep duration (≤ 7 hours and > 7 hours), ambient PM_2.5_ levels (μg/m^3^), skin color (White and Other), use of sun exposure protection (Yes and No).

**Table S8.** Associations between average sunlight exposure and elevated serum 25-hydroxyvitamin D concentrations (> 49.72 nmol/L) in the UK Biobank cohort.

| Outdoor duration | OR (95% CI) | *P* Value |
| --- | --- | --- |
|  |  |  |
| Average exposure time, hours |  |  |
| 2.5 | Reference |  |
| < 2.5 | 0.87 (0.84, 0.89) | < 0.001 |
| > 2.5 | 1.12 (1.09, 1.15) | < 0.001 |

Models were adjusted for age (< 65 years and ≥ 65 years), sex (Male and Female), ethnicity (White and Non-white), educational levels (College and Other levels), Body mass index (BMI) (kg/m2) (< 24, 24~27.9, ≥ 28), alcohol consumption (Yes and No), smoking status (Yes and No), household income (< £ 31,000 and ≥ £ 31,000), physical activity (Yes and No), dieting score (ranged from 0-7), sleep duration (≤ 7 hours and > 7 hours), ambient PM_2.5_ levels (μg/m^3^), skin color (White and Other), use of sun exposure protection (Yes and No). The mean of serum 25-hydroxyvitamin D concentrations was 49.72 nmol/L.

**Table S9.** Associations of average sunlight exposure with the risk of SLE, RA and gout stratified by serum 25-hydroxyvitamin D levels.

|  | Time spent in outdoor light (average) | | |  |  |
| --- | --- | --- | --- | --- | --- |
| Serum 25(OH)D, nmol/L | <= 2.5 hours | > 2.5 hours | | *P* for interaction | Adjusted *P* value |
|  | Reference | HR | *P* value |  |  |
| Systemic lupus erythematosus, SLE |  |  |  | 0.012 | 0.036 |
| <= 49.72 | 1 | 0.71 (0.46–1.08) | 0.109 |  |  |
| > 49.72 | 1 | 1.29 (0.86–1.94) | 0.216 |  |  |
| Rheumatoid Arthritis, RA |  |  |  | 0.695 | 1.000 |
| <= 49.72 | 1 | 1.08 (0.98–1.18) | 0.137 |  |  |
| > 49.72 | 1 | 1.11 (1.00–1.23) | 0.040 |  |  |
| Gout |  |  |  | 0.082 | 0.246 |
| <= 49.72 | 1 | 1.11 (1.02–1.20) | 0.012 |  |  |
| > 49.72 | 1 | 1.24 (1.13–1.36) | < 0.001 |  |  |

Models were adjusted for age (< 65 years and ≥ 65 years), sex (Male and Female), ethnicity (White and Non-white), educational levels (College and Other levels), Body mass index (BMI) (kg/m^2^) (< 24, 24~27.9, ≥ 28), alcohol consumption (Yes and No), smoking status (Yes and No), household income (< £ 31,000 and ≥ £ 31,000), physical activity (Yes and No), dieting score (ranged from 0-7), sleep duration (≤ 7 hours and > 7 hours), ambient PM_2.5_ levels (μg/m^3^), skin color (White and Other), use of sun exposure protection (Yes and No). *P* values for interaction were corrected for multiple comparisons using the Bonferroni method.

**Table S10**. Incidence proportions of SLE, RA, and gout according to ethnicity in the UK Biobank cohort.

| **Ethnicity** | SLE | | RA | | Gout | |
| --- | --- | --- | --- | --- | --- | --- |
|  | N | Cases, *n* (%) | N | Cases, *n* (%) | N | Cases, *n* (%) |
| White | 345671 | 306 (0.089%) | 344121 | 5276 (1.533%) | 344930 | 7124 (2.065%) |
| Non-White | 16607 | 28 (0.169%) | 16562 | 295 (1.781%) | 16601 | 334 (2.012%) |
| Missing | 1095 | 1 (0.091%) | 1094 | 20 (1.828%) | 1093 | 27 (2.470%) |

*Note.* RA: Rheumatoid arthritis, SLE: Systemic lupus erythematosus.
